# Local Evolutionary Patterns of Human Respiratory Syncytial Virus Derived from Whole-Genome Sequencing

**DOI:** 10.1128/JVI.03391-14

**Published:** 2015-01-21

**Authors:** Charles N. Agoti, James R. Otieno, Patrick K. Munywoki, Alexander G. Mwihuri, Patricia A. Cane, D. James Nokes, Paul Kellam, Matthew Cotten

**Affiliations:** a KEMRI-Wellcome Trust Research Programme, Kilifi, Kenya; b Public Health England, Salisbury, United Kingdom; c University of Warwick, School of Life Sciences and WIDER, Warwick, United Kingdom; d The Wellcome Trust Sanger Institute, Cambridge, United Kingdom; e Division of Infection Immunity, University College London, London, United Kingdom

## Abstract

Human respiratory syncytial virus (RSV) is associated with severe childhood respiratory infections. A clear description of local RSV molecular epidemiology, evolution, and transmission requires detailed sequence data and can inform new strategies for virus control and vaccine development. We have generated 27 complete or nearly complete genomes of RSV from hospitalized children attending a rural coastal district hospital in Kilifi, Kenya, over a 10-year period using a novel full-genome deep-sequencing process. Phylogenetic analysis of the new genomes demonstrated the existence and cocirculation of multiple genotypes in both RSV A and B groups in Kilifi. Comparison of local versus global strains demonstrated that most RSV A variants observed locally in Kilifi were also seen in other parts of the world, while the Kilifi RSV B genomes encoded a high degree of variation that was not observed in other parts of the world. The nucleotide substitution rates for the individual open reading frames (ORFs) were highest in the regions encoding the attachment (G) glycoprotein and the NS2 protein. The analysis of RSV full genomes, compared to subgenomic regions, provided more precise estimates of the RSV sequence changes and revealed important patterns of RSV genomic variation and global movement. The novel sequencing method and the new RSV genomic sequences reported here expand our knowledge base for large-scale RSV epidemiological and transmission studies.

**IMPORTANCE** The new RSV genomic sequences and the novel sequencing method reported here provide important data for understanding RSV transmission and vaccine development. Given the complex interplay between RSV A and RSV B infections, the existence of local RSV B evolution is an important factor in vaccine deployment.

## INTRODUCTION

Human respiratory syncytial virus (RSV) is a leading viral cause of severe respiratory infection during infancy and early childhood and among immunocompromised populations (1, 2). Globally, the virus is estimated to be responsible for 30 million episodes of acute lower respiratory tract infections (RTIs) and more than 50,000 deaths annually in children under 5 years of age (3). RSV infections throughout the world consistently occur as annual or biennial epidemics, and persons of all ages can be infected with diverse clinical outcomes ranging from mild upper RTIs to severe pneumonia or bronchiolitis (2, 4). A vaccine against RSV is not yet available (5). Careful analyses of RSV molecular epidemiology, evolution, and transmission are essential for defining the circulating viruses, for characterizing antigenic variation, and for tracking transmission patterns. The outcome of these studies can support new strategies for RSV control and vaccine use and development.

It has long been known that children suffer repeated RSV infections throughout life (6, 7). The ability of the virus to continue to infect previously exposed individuals is thought to be linked to an ability to bypass preexisting immune responses (8). Sequence variation in attachment (G) protein in consecutive years (9) is thought to be part of this mechanism; also, the global existence of two groups, A and B, and their alternating infection incidences may play a role (10, 11). The transmissibility of RSV group A (RSVA) is estimated to be slightly higher than that of RSV group B (RSVB) (12), and RSVA infections are more frequent than RSVB infections (12). An additional important feature of RSV infection is the apparently rapid global dispersion of new RSV variants (13). Indeed, genetically similar viruses cluster more by time than by location, suggesting rapid global movement of new variants (14). RSV molecular pathology and epidemiology have been reviewed in detail elsewhere (2, 4, 15, 16).

Historically, RSV molecular epidemiology has focused on the 900-bp region encoding the G protein (15, 17). The G protein together with the fusion (F) protein are important targets of human protective antibody responses (8, 15), with changes in this region thought to be driven by pressure to avoid host immune responses. Although studies of the sequence variability of RSV have concentrated on G gene variability, given the rapid infection pace but relatively low evolutionary rate of RSV, transmission studies over short periods require the stronger evolutionary signal provided by the full virus genome sequence (15,200 nucleotides [nt]: 11 open reading frames [ORFs] and noncoding regions). There is also a need to understand the nature of variation of immune targets other than the G protein. Advances in primer design, sequencing technology, and sequence assembly algorithms now allow full-genome sequencing for a number of viruses, including RSV (18–23), norovirus (24), and Middle East respiratory syndrome (MERS) coronavirus (25, 26).

The current work describes RSV genome evolution across a set of clinical samples collected from children who presented with severe RSV disease in a rural coastal Kenyan hospital using a novel RSV whole-genome sequencing (WGS) approach optimized for small amounts of clinical diagnostic samples. The sequence data provide an update of the genome-wide diversity of circulating RSV strains in this part of Kenya, including both RSVA and RSVB and the recently reemerged group B genotype GB3 (27). The novel genomes support previous conclusions on patterns of local RSV variation relative to global RSV diversity and reveal a significant difference in local evolution of RSVA versus RSVB.

## MATERIALS AND METHODS

### Primer design.

All RSV sequences available (August 2012) with lengths of >14,000 nt were collected and sorted by group, yielding 138 RSVA and 38 RSVB genomes. The sequences for each group were pooled and sliced into 33-nt strings with a 1-nt step size. The 33-mers were filtered to remove sequences with ambiguous nucleotides, and the frequency of each sequence within the set was determined. The 33-nt sequences were then trimmed to a calculated melting temperature (*T_m_*) of 58°C, discarding sequences mapping to human rRNA, with GC contents of <30% or >65%, or with a single nucleotide frequency of >60%. The RSV genome was divided into six 3-kb segments overlapping by 300 nt. All sequences were mapped to an RSVA or RSVB reference strain, and the two most frequent primers mapping within 300 nt of the end of each amplicon were selected. The reverse complement of the downstream sequences was prepared. To ensure amplification of the far ends of the genomes, two additional primers were included from the 5′- and 3′-terminal genomic regions. A summary of the primer sequences and their predicted target sequences across all known RSV genomes is presented in Table 1.

**TABLE 1:** Summary of RSV primers used in this study

Target	Primer	Sequence (5′ to 3′)	Strand	Position^*a*^	*T_m_* (°C)^*b*^	% with 0 MM^*c*^	% with 0–3 MM^*d*^
RSVA	rsvas	ACGCGAAAAAATGCGTACAAC	Plus	1	57.13	18.28	18.97
RSVA	rsva52	TGTGCATGTTATTACAAGTAGTGATATTTG	Plus	266	56.96	95.52	98.97
RSVA	rsva50	GCATGTTATTACAAGTAGTGATATTTGCC	Plus	269	57.51	95.17	98.97
RSVA	rsva117	ATAAGAGATGCCATGGTTGGTTTAAGA	Plus	2849	58.44	95.86	100.00
RSVA	rsva86	AAGAGATGCCATGGTTGGTTTAAGA	Plus	2851	58.43	95.86	100.00
RSVA	rsva175	TTCTCTTAAACCAACCATGGCATCT	Minus	2878	58.43	95.86	100.00
RSVA	rsva39	CTTCTCTTAAACCAACCATGGCATC	Minus	2879	58.22	95.86	100.00
RSVA	rsva1820	GCAGCATATGCAGCAACAATC	Plus	5207	56.95	93.79	98.97
RSVA	rsva1914	CAGCATATGCAGCAACAATCCAA	Plus	5208	58.32	93.10	98.62
RSVA	rsva1644	CAACTCCATTGTTATTTGCCCC	Minus	5674	56.05	89.66	100.00
RSVA	rsva1688	CAACTCCATTGTTATTTGCCCCA	Minus	5674	57.54	89.66	100.00
RSVA	rsva704	ATGTGTTGCCATGAGCAAACTC	Plus	7893	57.95	91.03	100.00
RSVA	rsva731	GCCATGAGCAAACTCCTCACT	Plus	7900	58.49	71.38	99.31
RSVA	rsva341	TTGTCAGGTAGTATCATTATTTTTGGCATG	Minus	8196	58.53	98.97	99.31
RSVA	rsva312	AGGATATTTGTCAGGTAGTATCATTATTTTTGG	Minus	8203	58.08	98.97	100.00
RSVA	rsva374	AAGAGAACTCAGTGTAGGTAGAATGTTT	Plus	10360	57.89	96.55	100.00
RSVA	rsva350	AGAACTCAGTGTAGGTAGAATGTTTG	Plus	10363	56.64	96.55	100.00
RSVA	rsva497	GCTTGATTGAATTTGCTGAGATCTGT	Minus	10620	58.44	95.52	100.00
RSVA	rsva539	ATGCTTGATTGAATTTGCTGAGATCTG	Minus	10622	58.68	95.52	100.00
RSVA	rsva1220	GATTGGGTGTATGCATCTATAGATAACAAG	Plus	12386	57.94	95.86	99.31
RSVA	rsva1232	ATTGGGTGTATGCATCTATAGATAACAAG	Plus	12387	57.17	95.86	99.31
RSVA	rsva364	TTATATATCCCTCTCCCCAATCTTTTTCAAA	Minus	13070	58.32	96.21	100.00
RSVA	rsva385	ATCAGTTATATATCCCTCTCCCCAATCTT	Minus	13075	58.46	96.21	100.00
RSVA	rsva4066	GTTGTATAACAAACTACCTGTGATTTTAATCAG	Minus	14983	57.95	88.97	99.31
RSVA	rsva5632	TAACTATAATTGAATACAGTGTTAGTGTGTAGC	Minus	15063	57.95	29.31	95.17
RSVA	rsvae	ACGAGAAAAAAAGTGTCAAAAACTAATA	Minus	15223	55.09	17.59	18.28
RSVB	rsvbs	ACGCGAAAAAATGCGTACTACA	Plus	1	57.56	43.14	43.14
RSVB	rsvb3	TGGGGCAAATAAGAATTTGATAAGTGC	Plus	44	58.58	48.04	54.90
RSVB	rsvb1021	GGGGCAAATAAGAATTTGATAAGTGCTATT	Plus	45	58.75	47.06	54.90
RSVB	rsvb33	ATATTAGGAATGCTCCATACATTAGTAGTTG	Plus	2777	57.21	88.24	100.00
RSVB	rsvb71	TAAGAGATGCTATGGTTGGTCTAAGAGA	Plus	2841	58.69	90.20	100.00
RSVB	rsvb50	AGTCTTGCCATAGCCTCTAACCT	Minus	2937	58.57	93.14	100.00
RSVB	rsvb95	CCATTTTTTCGCTTTCCTCATTCCTA	Minus	2963	58.14	95.10	100.00
RSVB	rsvb7884	AGTATATGTGGCAACAATCAACTCTG	Plus	5202	57.48	81.37	100.00
RSVB	rsvb7996	TATGTGGCAACAATCAACTCTGC	Plus	5206	57.70	81.37	100.00
RSVB	rsvb7442	GATGTGGAGGGCTCGGATG	Minus	5548	57.92	75.49	100.00
RSVB	rsvb7423	CCATGGTTATTTGCCCCAGATTTAAT	Minus	5662	57.87	77.45	99.02
RSVB	rsvb3762	AGAGGTCATTGCTTGAATGGTAGAA	Plus	7642	57.98	93.14	100.00
RSVB	rsvb3712	AAGAGCATAGACACTTTGTCTGAAATAAG	Plus	7762	57.89	77.45	100.00
RSVB	rsvb3652	GCTTATGGTTATGCTTTTGTGGATATCTAAT	Minus	8130	58.41	89.22	98.04
RSVB	rsvb3660	GCAATCATGCTTTCACTTGAGATCAA	Minus	8247	58.67	64.71	98.04
RSVB	rsvb32	AAGAAGAGTACTAGAGTATTACTTGAGAGATAA	Plus	10236	57.04	90.20	100.00
RSVB	rsvb52	AAATCCAAATCTTAGCAGAGAAAATGATAG	Plus	10412	56.70	96.08	100.00
RSVB	rsvb47	CCATGCAGTTCATCTAATACATCACTG	Minus	10673	58.13	90.20	99.02
RSVB	rsvb168	TGCATGTCTATATGTACATATTATTGTGACAAG	Minus	10746	58.25	91.18	99.02
RSVB	rsvb651	ATCGACATTGTGTTTCAAAATTGCATAAG	Plus	12640	58.40	81.37	100.00
RSVB	rsvb165	TTCAAAATTGCATAAGTTTTGGTCTTAGC	Plus	12653	58.06	88.24	100.00
RSVB	rsvb27	TTAATGAACATATGATCAGTTATATACCCCTCT	Minus	13088	57.88	79.41	100.00
RSVB	rsvb60	AACTTAAAACTGTGACAGCCTTTTATTCT	Minus	13325	58.08	89.22	100.00
RSVB	rsvb1199	ATAGTACACTACCTGTTATTTTAATCAGCTTCT	Minus	14977	58.56	88.24	100.00
RSVB	rsvb989	TATAGTACACTACCTGTTATTTTAATCAGCTTC	Minus	14978	57.57	88.24	100.00
RSVB	rsvbe	ACGAGAAAAAAAGTGTCAAAAACTAATGT	Minus	15216	57.47	5.88	6.86

a Primer mapping position in RSVA (GenBank accession number FJ948820) or RSVB (GenBank accession number JQ582843).

b *T_m_* (melting temperature) calculated using a Python script that approximates the method of Breslauer et al. (51).

c Percentage of full-length RSVA genomes (*n* = 290) or full-length RSVB genomes (*n* = 102) showing perfect homology to primer, i.e., 0 mismatches (MM).

d Percentage of full-length RSVA genomes (*n* = 290) or full-length RSVB genomes (*n* = 102) showing the target sequence for the primer with up to 3 mismatches.

### Clinical samples.

Viral nucleic acid for sequencing was extracted from RSV-positive clinical specimens (nasopharyngeal swabs [NPS] or washes) collected from children under 5 years old admitted to the Kilifi District Hospital (KDH) with severe or very severe pneumonia between 2002 and 2012. RSV infection was diagnosed with an indirect immunofluorescence antibody technique (IFAT; Light Diagnostics). Details of the study that provided the samples sequenced in this study have been previously provided (28). Informed consent was obtained from a parent or guardian on behalf of each child before specimen collection, and the KEMRI Ethics Review Committee approved all protocols. Additional details on the samples are provided in Table 2.

**TABLE 2:** Details for samples used in this study

MiSeq	Age (mo)	Sample date (day-mo-yr)	Group	Length (nt)^*a*^	Coverage^*b*^	Present in G set^*c*^	Present in F set^*d*^	GenBank no.^*e*^	ENA no.^*f*^
10028_10	0	07-Jan-02	A	9,346	6,401	Yes		KP317918	ERR323212
10028_11	6	27-Apr-02	A	7,091	10,370			KP317940	ERR323213
10028_12	6	28-Jan-03	A	9,776	5,692		Yes	KP317955	ERR323214
11866_65	5	13-Feb-03	A	12,151	7,347	Yes	Yes	KP317949	ERR438932
11865_75	8	24-Mar-04	A	14,985	12,283	Yes	Yes	KP317956	ERR438910
10891_50	6	21-Jan-05	A	5,396	3,554	Yes	Yes	KP317948	ERR376407
10891_56	0	02-Feb-05	A	5,396	2,369	Yes	Yes	KP317924	ERR376413
9696_45	14	20-Feb-06	A	14,778	3,830	Yes	Yes	KP317944	ERR303303
10891_57	1	23-Feb-06	A	14,841	4,640	Yes	Yes	KP317942	ERR376414
10891_58	0	29-Mar-06	A	8,864	6,016			KP317943	ERR376415
10891_59	3	04-Jan-07	A	11,496	5,316		Yes	KP317937	ERR376416
10891_60	1	05-Jan-07	A	14,791	4,454	Yes	Yes	KP317926	ERR376417
10891_51	0	07-Mar-08	A	14,967	4,882	Yes	Yes	KP317933	ERR376408
10891_52	11	17-Mar-08	A	5,636	1,201	Yes		KP317931	ERR376409
10899_38	1	22-Feb-09	A	14,854	8,478	Yes	Yes	KP317950	ERR381723
10899_40	4	26-Jan-10	A	10,113	13,351		Yes	KP317916	ERR381725
10899_41	18	10-Feb-10	A	14,713	12,405	Yes	Yes	KP317935	ERR381726
11864_54	3	29-Apr-10	A	14,716	7,071	Yes	Yes	KP317936	ERR438905
11862_33	3	26-Aug-10	A	14,719	8,961	Yes	Yes	KP317921	ERR438868
11864_53	1	25-Mar-11	A	14,735	6,891	Yes	Yes	KP317951	ERR438904
11862_28	28	13-Apr-11	A	15,214	10,434	Yes	Yes	KP317920	ERR438864
11862_29	4	23-Mar-12	A	14,950	12,922	Yes	Yes	KP317953	ERR438865
11862_32	14	30-Apr-12	A	7,197	6,180			KP317947	ERR438867
9697_16	10	06-Jul-02	B	15,040	5,419	Yes	Yes	KP317939	ERR303322
9697_10	8	13-Jan-03	B	9,790	6,853		Yes	KP317930	ERR303316
10140_1	46	02-Apr-04	B	12,034	12,174	Yes	Yes	KP317919	ERR331021
9697_7	10	22-Dec-04	B	15,080	4,480	Yes	Yes	KP317925	ERR303313
9697_6	2	25-Dec-04	B	14,998	6,523	Yes	Yes	KP317954	ERR303312
9697_5	1	27-Jan-06	B	15,234	3,682	Yes	Yes	KP317917	ERR303311
9465_10	23	27-Feb-09	B	14,995	16,190	Yes	Yes	KP317938	ERR303268
9465_11	31	13-Feb-10	B	15,004	11,722	Yes	Yes	KP317941	ERR303269
9465_12	22	06-Apr-10	B	15,260	14,855	Yes	Yes	KP317932	ERR303270
9465_6	17	09-May-10	B	15,333	13,719	Yes	Yes	KP317952	ERR303264
9465_7	3	01-Feb-11	B	15,233	14,182	Yes	Yes	KP317927	ERR303265
9465_8	2	14-Apr-11	B	15,323	15,367	Yes	Yes	KP317945	ERR303266
9465_9	1	08-Jul-11	B	15,237	14,709	Yes	Yes	KP317928	ERR303267
9465_3	8	14-Jan-12	B	14,995	12,378	Yes	Yes	KP317946	ERR303261
9465_1	19	13-Feb-12	B	15,233	12,994	Yes	Yes	KP317934	ERR303259
9465_4	14	01-Mar-12	B	15,179	14,802	Yes	Yes	KP317923	ERR303262
10911_9	1	23-Mar-12	B	14,977	12,504	Yes	Yes	KP317929	ERR376442
9465_2	5	16-May-12	B	14,941	12,906	Yes	Yes	KP317922	ERR303260

a Final sequence length obtained from *de novo* assembly of short read data (see Materials and Methods).

b Coverage calculated by mapping all reads to final assembled contig. Coverage was calculated as the number of mapped reads/(length of the genome fragment/129).

c Samples yielding sufficient sequence for G region analysis (Fig. 5).

d Samples yielding sufficient sequence for F region analysis (Fig. 5).

e The final genome data were deposited in GenBank with the indicated accession numbers.

f Short-read data available at European Nucleotide Archive (http://www.ebi.ac.uk/ena).

### RNA extraction, RT, and PCR.

Viral RNA was extracted with the QIAmp extraction kit (Qiagen, United Kingdom) from a starting NPS specimen volume of 140 μl and final elution volume of 60 μl. Reverse transcription (RT) of RNA molecules was performed with the forward primers for each of the six amplicons. A separate RT reaction was performed for each amplicon. Typically, the 20-μl reaction mixture contained 2 μl of sample RNA. A 5-μl aliquot of the resulting cDNA was used in each 25-μl PCR mixture. The PCR mixture was incubated at 98°C for 30 s, followed by 40 cycles of 98°C for 10 s, 53°C for 30 s, and 72°C for 3.0 min and a final extension of 72°C for 10.0 min. Following PCR, aliquots of the products were run on a 0.6% agarose gel to monitor amplification success, and the products from the 6 reactions for each sample were pooled for Illumina library preparation.

### Deep sequencing.

Sequencing of the pooled amplicons was performed with Illumina MiSeq. Samples were multiplexed at 15 to 20 per MiSeq run and processed as paired-end reads (2 × 149 nt), generating approximately 1.5 million reads per sample. Raw sequence data were processed with QUASR (29) to remove low-quality (< median Phred 35) and adapter-containing reads, and *de novo* assembly with SPAdes (30) was performed. RSV contigs were identified by BLASTN analysis, and low-coverage contigs were excluded. Where necessary, partial but overlapping genome contigs were combined using Sequencher (v5.2.4). All final viral genomes were examined for appropriate assembly based on length and the presence of the expected intact RSV open reading frames.

### Protein changes.

After sorting by virus group (RSV group A or B), the genomic region under investigation was translated, the protein sequence was aligned using MAFFT (31), and protein differences from the consensus sequence of the group were visualized and quantitated using Python scripts.

### Reference data set.

A comprehensive RSV genome data set was generated from the GenBank database using as a starting set all reported RSV genomes. The search was conducted on 28 September 2014 using the search term “txid11250 [Organism]) AND 13500[SLEN]: 17000[SLEN].” Genomes with multiple ambiguous bases, lacking country of detection or date of collection (year), or from patent depositions were excluded. The newly sequenced Kilifi RSV genomes for each group were combined with those from GenBank in the subsequent analysis. Thinned representative reference sets were prepared by using the usearch algorithm (32).

### Phylogenetic analyses.

Phylogenetic trees of the genome sequences and selected genomic regions were constructed using the Bayesian methods in MrBayes program v3.2.1 (http://mrbayes.sourceforge.net/index.php) under the general time reversible model of evolution. RSVA and RSVB were analyzed separately using both the total data set and the thinned data sets. The viruses within the groups were assigned to genotypes based on the clustering pattern of the G ORF portion sequences with reference sequences representative of the previously described RSV genotypes: for RSVA, strains representing GA1-7, SAA1, and ON1, and for RSVB, strains representing GA1-4, SAB1-SAB4, and BA (11, 33–35). Phylogenetic trees were visualized in FigTree v1.4.2.

### Evolutionary analyses.

Nucleotide substitution rates and estimates for time to most recent common ancestor (tMRCA) were obtained from the usearch-thinned data sets, using uclust to remove genomes closer than ID 0.99 (32). The rates and tMRCA estimates were calculated in BEAST v1.7.5 (36) both for full genomes and for the individual ORFs.

### Nucleotide sequence accession numbers.

The final set of RSV sequences was deposited in GenBank with the following accession numbers: KP317916 to KP317956.

## RESULTS

**FIG 1:**
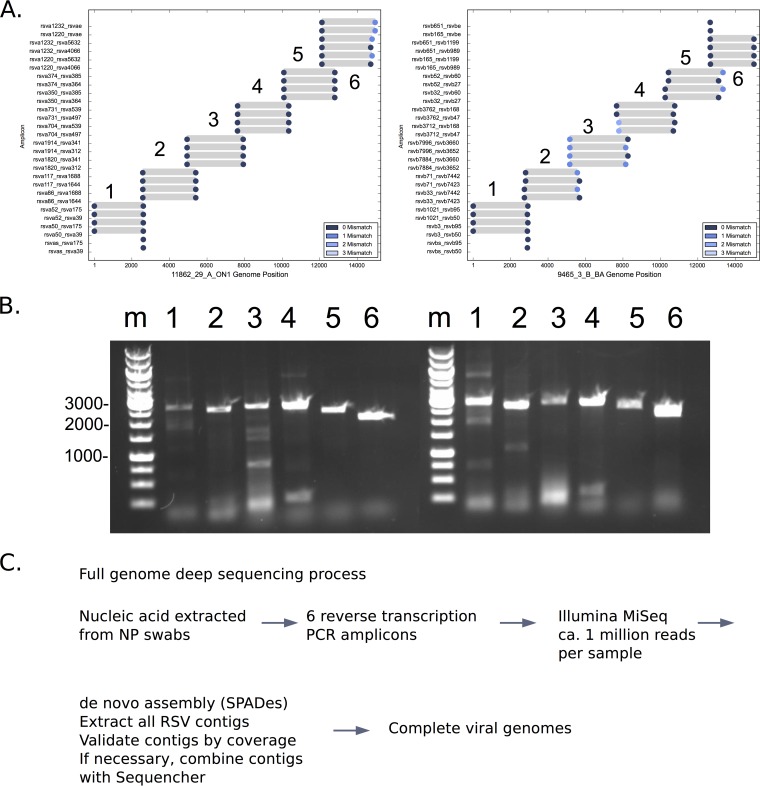
(A) PCR primer target sites in RSVA and RSVB. The primer target sequences in representative RSVA (left) and RSVB (right) viruses were determined. Circular markers indicate positions of primer target sites in the test genome color-coded by number of mismatches with the primer; gray bars indicate lengths and positions of the predicted products. (B) Two examples of reverse transcription-PCR function. The DNA products of reverse transcription and PCR amplification of two samples were resolved by agarose gel electrophoresis and visualized by ethidium bromide staining. Sizes of some of the molecular size markers (in base pairs) are indicated to left of the gel. Lane m, molecular size markers; lanes 1 to 6, individual 2- to 3-kb RSV amplicons 1 to 6, respectively. (C) Flowchart of the RSV sequencing process.

Two sets of reverse transcription and PCR primers were selected from all available RSVA and RSVB genomic sequence data based on frequency, location, and predicted PCR function (see Table 1 for further details). The general pattern of primer sites and the locations of primer targets in RSVA and RSVB genomes are shown in Fig. 1A. Actual PCR results are shown in Fig. 1B for RSVA and RSVB samples, with PCR products of the expected size obtained for all 6 amplicons. These primers were used as part of a deep-sequencing process for RSV combining the full cDNA preparation and genome amplification, deep sequencing with Illumina MiSeq, and *de novo* assembly (Fig. 1C) to generate 27 complete or nearly complete genomes (11 group A and 16 group B; median length, 14,990 nt; range, 14,666 to 15,232 nt). An additional number of samples yielded RSV contigs of >5,000 nt in length, and these were also retained for further analysis. A summary of the genomic sequences in this study is provided in Table 2.

### RSV global phylogenetic clustering and placement of Kilifi genomes.

**FIG 2:**
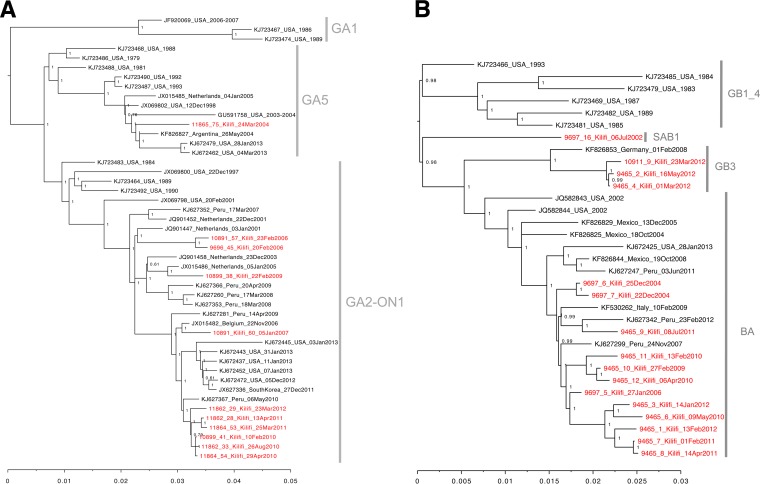
Phylogenetic analysis of the Kilifi RSVA and RSVB genomes. (A) MrBayes tree of representative global RSVA genome sequences together and the 11 novel Kilifi RSVA genome sequences. (B) MrBayes tree of representative global RSVB genome sequences and the 16 novel Kilifi RSVA genome sequences. Trees were inferred using the Bayesian methods in MrBayes (http://mrbayes.sourceforge.net/index.php) under the GTR model of evolution. The numbers next to the branches indicate the posterior probabilities. The Kilifi taxa are indicated in red font. Thinned global reference sets for RSVA and RSVB were prepared from all available RSV genomes clustering at 0.99% identity using uclust (32). See Materials and Methods for additional details.

The 27 Kilifi genomes were combined with RSVA and RSVB genomes from 16 countries from specimens collected between the years 1981 and 2013 (see Materials and Methods). The phylogenetic clustering is shown in Fig. 2A (RSVA) and B (RSVB). RSVA forms 3 major clades: GA1 (including strains only from the United States), GA5 (with U.S. and global strains), and a clade with both GA2 and the ON1 viruses with a 72-nucleotide duplication in the G ORF (33), which included nearly all of the new Kilifi RSVA genomes (GA2_ON1). Multiple subclusters showing temporal clustering were detected within each of these clades.

Four clades were designated for the RSVB genomes, with BA containing the majority of the Kilifi sequences (Fig. 2B). Clade GB1_GB4 included viruses detected in the United States between 1983 and 1991. Clades SAB1, GB3, and BA included viruses from multiple countries, including the Kilifi RSVB genomes. Similar to that for RSVA, the clustering was more temporal than geographical. Notably, the BA (Buenos Aires) clade viruses are characterized by the presence of a 60-nucleotide duplication within the G ORF. The 4 viruses within clade GB3 (3 from Kilifi and 1 from Germany) lacked the 60-nucleotide duplication. Neither RSVA nor RSVB genomes from Kilifi showed a monophyletic grouping. Instead, the Kilifi genomes were dispersed throughout the observed RSV evolution, clustering with contemporaneous genomes from the other countries. The phylogenetic tree topologies arising from whole-genome and G protein ORF sequences were highly similar (data not shown).

### Comparison of genomes of viruses with identical G protein ORFs.

**FIG 3:**
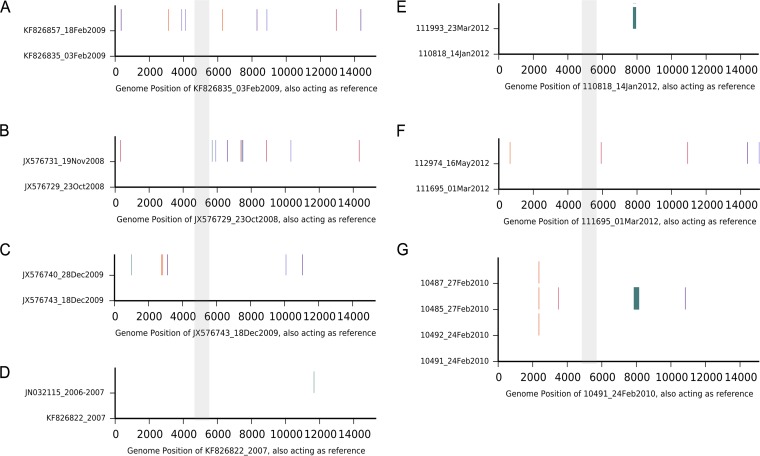
Comparison of RSVB genomes with identical G regions. Each panel represents a genome nucleotide alignment of RSVs that had identical G gene sequences. The G protein ORF portions of the genomes are highlighted gray across the panels and were identical. The vertical lines indicate where there are nucleotide substitutions occurring outside the G gene region between the genomes. The blue blocks indicate a gap in the sequence.

One motivation for developing full-genome methods was to increase the sensitivity for tracking RSV across short-term transmission chains. We asked if viruses identical in their G gene regions had differences elsewhere in their genomes. All RSV genomes (both GenBank or in the new data presented here) with identical G regions were identified, and the number of changes outside the G region were determined. Of 7 sets of viruses with identical G regions, all showed at least 1 but up to 9 nucleotide differences across the full genome (Fig. 3). This increased resolution will be important in future studies examining RSV household transmission patterns to identify who acquires infection from whom.

### Estimation of RSV tMRCA and evolutionary rates.

**FIG 4:**
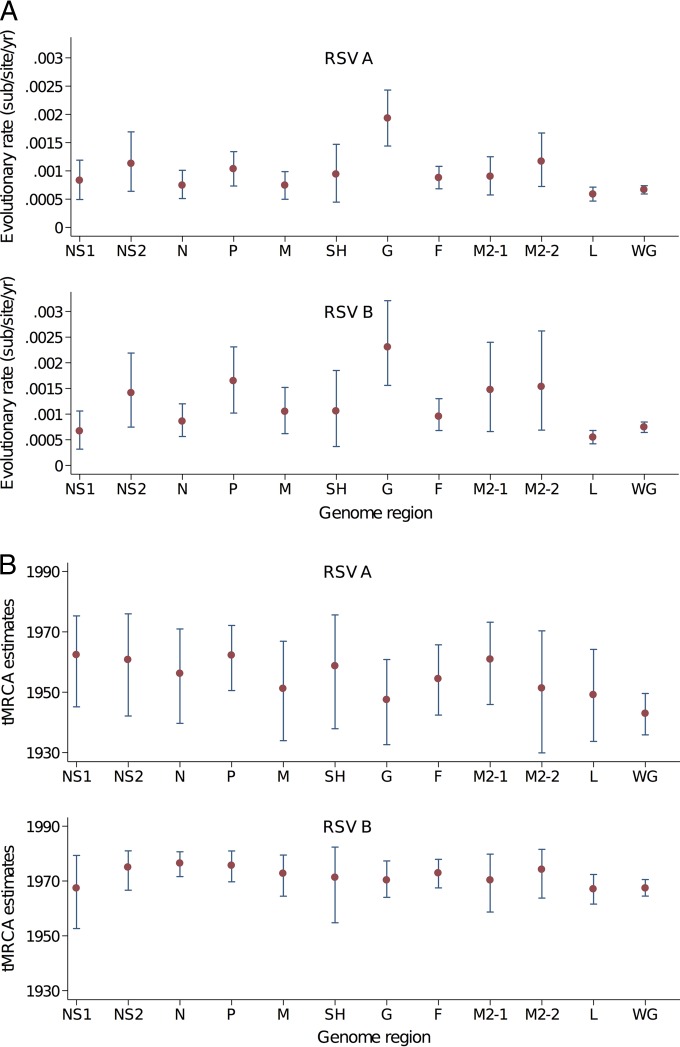
(A) Estimates of the nucleotide substitution rates for RSVA and RSVB in the individual ORFs and for the whole-genome sequence. (B) Estimates of tMRCA for RSVA and RSVB for the individual ORFs and for the whole-genome sequence. The analysis was undertaken using the usearch-thinned data sets (37 genome sequences for RSVA and 23 sequences for RSVB). The analysis was performed with BEAST (36).

Previous data on RSV evolution are largely derived from the G protein coding region. The full genomes generated in this study were combined with the GenBank reference data set, and these allowed an estimation of the global nucleotide substitution rates and the time to most recent common ancestor (tMRCA) for all the recently sequenced RSVA and RSVB viruses. These estimates were calculated for the different ORFs and the whole-genome sequences (Fig. 4). The whole genomes provided more precise estimates of the MRCA, as observed from the interval of lower and upper 95% highest posterior density (HPD) compared to individual ORF data for the same set of viruses (Fig. 4B).

The G protein ORF showed the highest nucleotide substitution rates for both RSVA and RSVB (Fig. 4A). Elevated changes in G and M2-2 were observed previously using RSV full genomes from U.S. and European cohort data (21). Similar to the MRCA estimates, the whole-genome estimates for the evolutionary rates showed narrower confidence intervals than those from the individual ORFs. The two regions considered for vaccine targets, G and F, show a strikingly wide difference in rate, and this may be important for selecting conserved vaccine targets.

### Changes in G and F coding regions, comparing local and global viruses.

**FIG 5:**
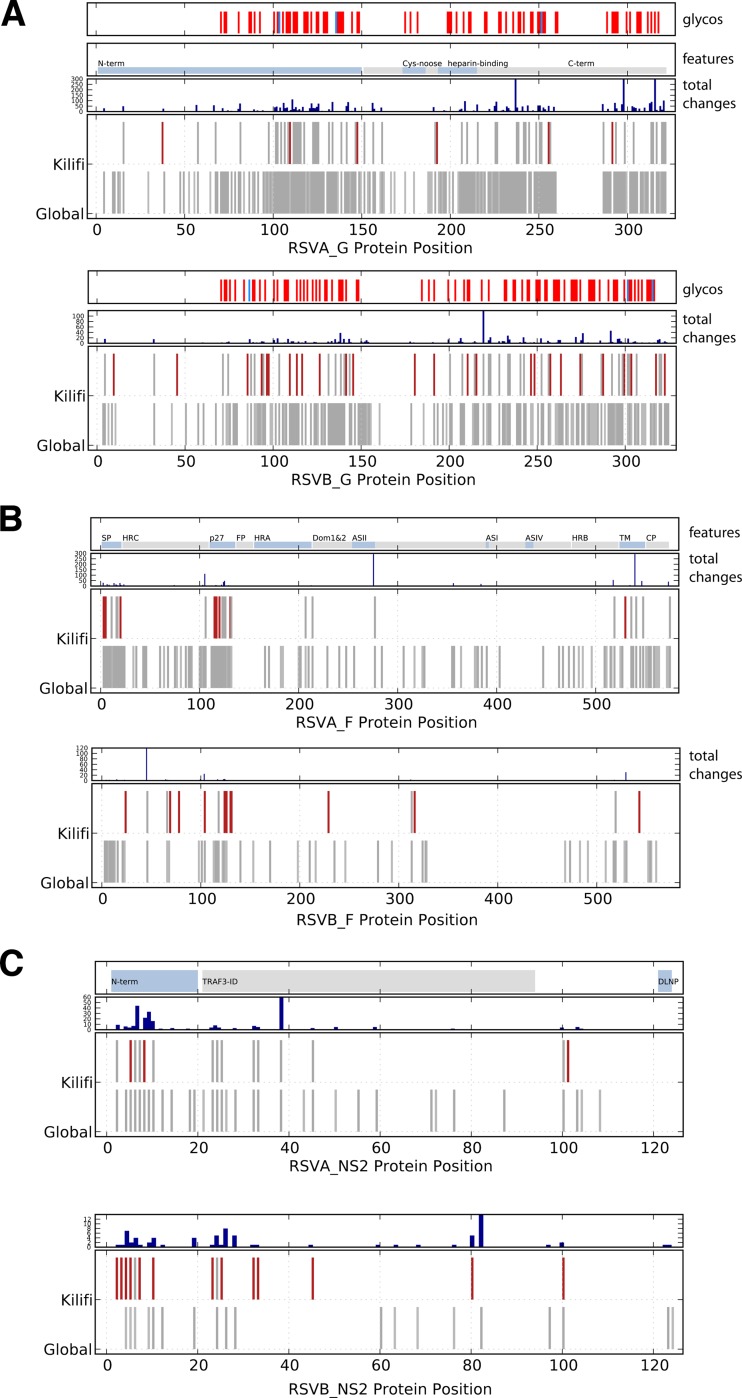
Kilifi versus global changes in the G, F, and NS2 proteins. (A) Kilifi compared to global G protein changes. For each group, the G protein sequences were identified as Kilifi or non-Kilifi (global) and aligned, and a consensus amino acid sequence was generated (at 60% level). The first portion shows the positions of O-linked (red) and N-linked (blue) glycosylation sites, the second portion shows general features of the G protein, and the third portion shows total changes (Kilifi plus global) at each position. The fourth portion shows amino acid differences in each G sequence from the consensus. Amino acid changes observed only in Kilifi are marked in red, and changes observed either globally or in the Kilifi are marked in gray. Gaps are not indicated. N-linked and O-linked glycosylation sites were determined using NetNGlyc 1.0 and NetOGlyc 3.1 (46–48). (B) Kilifi versus global F protein changes. Changes in F protein were determined and are depicted as in panel A. Known motifs of the F protein (49) include signal peptide (SP), heptad repeat C (HRC), 27-mer fragment (p27), putative fusion peptide (FP), heptad repeat A (HRA), domains 1 and 2 (Dom1&2), heptad repeat B (HRB), transmembrane domain (TM), and cytoplasm domain (CP). Antigenic sites I, II, and IV (ASI, ASII, and ASIV) are sites of neutralizing antibody binding (40, 50). (C) Kilifi versus global NS2 protein changes. Changes in NS2 protein were determined and are depicted as in panel A. Known motifs of the NS2 protein include the TRAF3-interacting domain (TRAF3-ID) and C-terminal tetrapeptide sequence (DLNP) (43).

An important consideration for vaccine development is how representative a vaccine strain is for locally circulating viruses. The transmission patterns of a virus, the evolutionary rate of the virus, and patterns of human movement can strongly influence how quickly global strains reach a rural location. To address this important issue, the amino acid changes encoded by the RSV coding sequences observed in Kilifi were compared to the amino acid changes observed for all known RSV genomes from other parts of the world (Fig. 5; Table 3).

**TABLE 3:** Kilifi versus global evolution

Protein	No. of distinct changes for all Kilifi and global viruses	No. of distinct changes in Kilifi viruses	No. (%) of distinct changes unique to Kilifi viruses^*a*^
RSVA G	409	68	7 (11.8)^*b*^
RSVB G	299	70	30 (42.9)
RSVA F	200	45	9 (22.2)^*c*^
RSVB F	81	19	13 (79.3)
RSVA NS2	73	18	3 (16.7)^*d*^
RSVB NS2	38	16	13 (81.3)

a Number of distinct amino acid changes observed in Kilifi and not in other parts of the world. “Distinct changes” means that the set of changes is reduced to a unique set with multiple occurrences of a change counted only once.

b The *P* value for Fisher's exact test was <0.01 for the number of location-specific distinct changes compared to total distinct changes for RSVA versus RSVB.

c The *P* value for Fisher's exact test was <0.05 for the number of location-specific distinct changes compared to total distinct changes for RSVA versus RSVB.

d The *P* value for Fisher's exact test was <0.05 for the number of location-specific distinct changes compared to total distinct changes for RSVA versus RSVB.

A large percentage of the changes observed in the RSVA G protein were also observed globally, with 88% of the changes seen in Kilifi RSVA G also observed in other parts of the world (Table 3). The Kilifi RSVB viruses appeared to have more local evolution, with only 60% of the observed changes in G shared with global viruses. With reference to the F protein, for Kilifi RSVA viruses, 80% of the observed changes were also found globally, while the Kilifi RSVB viruses showed a higher degree of local evolution, with only 20% of the observed changes specific to Kilifi viruses seen in other locations. To determine if this local evolution of RSVB was observed at other sites, the sequence data were stratified to other locations (the United States, Argentina, and Peru), but no significant local patterns were observed. This suggests that the isolation of the Kilifi site was more pronounced than for other sites. Alternatively, this may reflect more intense sampling of RSVB within a limited area.

The RSV envelope proteins are heavily glycosylated. More than 50% of the G protein mass can be carbohydrate (37), and the potential O-linked glycosylation sites (serine or threonine) comprise up to 30% of the G protein amino acid sequence (38). Changes toward or away from asparagine can be associated with a change in the overall glycosylation of the protein and could be associated with adaptive change to local immune responses. The G protein is subject to heavy O-linked glycosylation in the variable regions, with modification frequently on serine or threonine residues in the vicinity of a proline residue (Fig. 5A). Nearly half of the observed changes in the in G protein affect S, T, or P residues (RSVA GA2 37/81 and RSVB 100/241). This is apparent when potential N- and O-linked glycosylation sites are marked on the G protein region (Fig. 5) and is also facilitated by the single nucleotide changes that distinguish codons for these three amino acids.

In the RSVA G proteins, an N237D polymorphism observed in many of the viruses is within the site NTT and would remove a predicted N-linked glycosylation site. Tan et al. (22) also noted that the RSVA-GA2 group showed a frequent change in two predicted glycosylation sites (N237D and S242N). Within the RSVB viruses, 3 of 15 amino acid changes involve asparagine, but none of these changes are predicted N-linked glycosylation sites (Asn-Xaa-Ser/Thr). Changes in N-linked glycosylation areas are known to effect binding of human convalescent-phase sera to peptides (39).

The RSV F protein contains only 10 to 20% of its mass as carbohydrate, and this is attached exclusively via N-glycosidic bonds (37). For the RSVA viruses, 5 of the protein changes are to or away from asparagine. In RSVB, 3 changes involve asparagine; however, none of these are within predicted glycosylation sites. Many polymorphisms were observed in the F protein p27 domain (Fig. 5B). This peptide is likely to serve as a spacer that is freed by cleavage during F maturation and is not found in the mature protein. The large number of changes may simply reflect the disposable nature of this sequence (40).

The NS2 protein may be important in modulating host innate immune responses (41–43) and may influence movement of infected cells (44). The NS2 showed an elevated level of evolutionary rate (Fig. 4), consistent with a protein interacting with polymorphic host target proteins. Monitoring the local versus global protein changes in NS2 revealed multiple changes occurring in the amino-terminal domain and a portion of the domain important for TRAF3 interactions (43). The majority of changes in the Kilifi RSVA NS2 proteins were also observed in other parts of the world; however, the RSVB NS2 protein showed a significantly high degree of variation only observed in the Kilifi viruses (Fig. 5C).

## DISCUSSION

The current work presents a functional approach for community-wide monitoring of RSV whole-genome genetic diversity suitable for detailed transmission studies. A challenge with deep sequencing of large sample sets of RNA viruses is the design of amplification primers. Traditionally, PCR primers were designed using alignments of sequences from the target virus; previous RSV studies with dideoxy sequencing used a greater number of tiled amplicons (2, 24, 25, 38) to cover the whole genome. With larger and more diverse sets, the alignment step becomes problematic. The approach described here bypasses the alignment step and was tailored for deep-sequencing methods. The RSV method uses only 6 amplicons to reduce the amplification costs and the required amount of input RNA. Although two primer sets were designed for RSVA and RSVB, the two sets can be pooled to simplify processing of samples of unknown RSV subtype. The computational methods used for primer selection facilitates updating of the primer sets as additional RSV genome sequence data become available. Frequent updating of these primer sets will help avoid sequence bias that could occur using antiquated primer sets. It is also important that the new full genomes reported here were assembled using *de novo* assembly methods. Although reference-based methods for assembling genomes from short-read data are rapid and less memory intensive, reference-based methods fail if a close reference genome is not available. The method presented here determines virus genomic sequences directly from patient material and shows sensitivity similar to that of traditional sequencing methods, but it avoids the potential virus selection that may occur if samples are first passaged through cell culture.

The 27 novel Kilifi RSV genomes (11 RSVA and 16 RSVB) generated in this study were used to assess local versus global RSV variety. Similar to the patterns previously observed with G ORF, the full genomic phylogenetic analysis confirmed that Kilifi genomes were interspersed with genomes from other countries, with rapid appearance of variants in Kilifi soon after they are first observed in other parts of the world (45). Kilifi RSV strains are similar to strains that circulate in other regions of the world and reveal only limited local evolution. Phylogenetic clustering appeared to be more influenced by time of virus sample collection than by geographical location, suggesting a fairly rapid global spread of novel RSV variants. It should also be noted that the similarity of the overall topology of phylogenetic trees from whole genomes and G sequences is encouraging and indicates that although full-genome sequences are most useful for detailed transmission studies, the relationships determined with the G region is similar to the patterns observed with the full-genome sequences.

The availability of full genomes allowed a comparison of estimates of the tMRCA of the Kilifi RSV strains. The obtained tMRCAs were broadly similar, although the higher evolutionary rates of the G region lead, as expected, to slightly later tMRCAs. The estimates based on the entire genomes lead to earlier dates and more discrete confidence intervals than estimates from specific genomic regions. Similar observations were made by Tan et al. (22).

Our comparison of genomes determined to be identical in the G region found nucleotide substitutions elsewhere in their genomes. The genomes with identical G regions invariably were from the same geographical region and over the same epidemic, the sample collection date interval ranging from a few days to months. This observation suggests that nucleotide substitution in the RSV genome in the short term is random, i.e., not concentrated in the regions that appear the most variable in the long term, and supports the use of whole-genome sequencing for monitoring viral transmission chains.

The observed sites of change in the G and F proteins were frequently in exposed regions of the proteins; several involved glycosylation site changes suggestive of immune evasion. In addition, similar to previous reports, the NS2 (Fig. 5B) and M2-2 protein coding regions (not shown) were observed to change at rates higher than that for the full genome. Although these changes could be simply the allowed changes of unconstrained proteins, it is also possible that these sites are important for interacting with the host and may be under some pressure to change. Unfortunately, the sequence data set generated in this study was too small to provide statistically supported evidence of positive selection, but future studies with larger data sets will be facilitated by these methods.

The availability of a collection of RSV genome sequences from a single African location allowed a comparison of local versus global RSV evolution patterns. Important for vaccine design, the RSVA variants observed in a small region of Kenya appear to be in equilibrium with global variants. The same was not observed for RSVB. Possibly, RSVB variants may spread less efficiently, with a higher fraction of variants observed to be specific for Kilifi and not detected in other parts of the world. This pattern is consistent with RSVB as a less transmissible infection than RSVA (4, 12). However, there are fewer global sequences available for RSVB, so while the Kilifi RSVB variants appear to be unique, this could be a consequence of less surveillance and documentation of RSVB variation globally. Future work will help clarify this phenomenon, as it may have strong consequences on the efficacy of any RSV vaccine used locally.
